# Health care workers’ perceptions of episiotomy in the era of respectful maternity care: a qualitative study of an obstetric training program in Mexico

**DOI:** 10.1186/s12884-021-04022-x

**Published:** 2021-08-12

**Authors:** Rodrigo Garcia-Cerde, Pilar Torres-Pereda, Marisela Olvera-Garcia, Jennifer Hulme

**Affiliations:** 1 Departamento de Salud Reproductiva (Department of Reproductive Health), Centro de Investigación en Salud Poblacional (Center for Research in Population Health), Instituto Nacional de Salud Pública de México (National Institute of Public Health of Mexico), Av. Universidad 655, Col. Sta. Maria Ahuacatitlán. Cp, 62100 Cuernavaca, Morelos Mexico; 2 Dirección de Investigación en Equidad para la Salud (Health Equity Research Department), Centro de Investiación en Sistemas de Salud (Center for Health Systems Research), Instituto Nacional de Salud Pública de México (National Institute of Public Health of Mexico), Av. Universidad 655, Col. Sta. Maria Ahuacatitlán. Cp, 62100 Cuernavaca, Morelos Mexico; 3 Department of Family and Community Medicine, University of Toronto, Toronto General Hospital, University Health Network, 200 Elizabeth Street, R. Fraser Elliott Building, Ground Floor, Room 480, Toronto, ON M5G 2C4 Canada

**Keywords:** Qualitative research, Episiotomy, Respectful maternity care

## Abstract

**Background:**

Episiotomy in Mexico is highly prevalent and often routine - performed in up to 95% of births to primiparous women. The WHO suggests that episiotomy be used in selective cases, with an expected prevalence of 15%. Training programs to date have been unsuccessful in changing this practice. This research aims to understand how and why this practice persists despite shifts in knowledge and attitudes facilitated by the implementation of an obstetric training program.

**Methods:**

This is a descriptive and interpretative qualitative study. We conducted 53 pre and post-intervention (PRONTO© Program) semi-structured interviews with general physician, gynecologists and nurses (*N* = 32, 56% women). Thematic analysis was carried out using Atlas-ti© software to iteratively organize codes. Through interpretive triangulation, the team found theoretical saturation and explanatory depth on key analytical categories.

**Results:**

Themes fell into five major themes surrounding their perceptions of episiotomy: as a preventive measure, as a procedure that resolves problems in the moment, as a practice that gives the clinician control, as a risky practice, and the role of social norms in practicing it. Results show contradictory discourses among professionals. Despite the growing support for the selective use of episiotomy, it remains positively perceived as an effective prophylaxis for the complications of childbirth while maintaining control in the hands of health care providers.

**Conclusions:**

Perceptions of episiotomy shed light on how and why routine episiotomy persists, and provides insight into the multi-faceted approaches that will be required to affect this harmful obstetrical practice.

## Background

In Mexico, childbirth is largely medicalized, with obstetrical procedures representing 45.2% of all medical interventions [1]. There are important advantages to high (95%) coverage of facility births in Mexico [2], but high perinatal morbidity (14.3%) suggest ongoing, major gaps in quality of care [3–5]. The relationship between low quality of health services and maternal and infant morbidity and mortality is well established [6, 7]. Miller and colleagues [7] describe these two extremes in maternity care as “too little, too late” (TLTL) where care is unavailable, unsafe, or available too late to help; and “too much, too soon” (TMTS), where the overutilization of interventions which can otherwise be lifesaving can cause harm when overused. TMTS includes routine episiotomies and other inappropriate interventions [8, 9]. Both TMTS and TLTL can co-exist, particularly in middle-income countries like Mexico [7].

Episiotomy is one of the most common obstetrical procedures [10]. It is the surgical incision of the perineum and posterior vaginal wall during the second stage of labour [11]. The complications – as compared to allowing the perineum to tear naturally - include an increased risk of anal sphincter injuries [12], persistent pain, pelvic floor defects, urinary and rectal incontinence, dyspareunia [10], the risk of tearing in the next delivery, and a longer recovery period [12, 13].

The prevalence of episiotomy in Mexico ranges from 41.8% in the state of Oaxaca to 77.2% in Mexico City - where up to 95% of primiparous women receive an episiotomy [14]; these data seem to reflect the routine practice of episiotomy despite ‘knowing’ about the complications [15, 16]. These figures are much higher when we compare them to the prevalence of episiotomy in the United States (25%) and Europe (30%) [17].

Scientific evidence and national norms and guidelines all support the selective use of episiotomy [18, 19]. The World Health Organization (WHO) recommends against routine episiotomy, and acknowledges that there is no evidence to support routine episiotomy in modern obstetrics [20, 21]. The American College of Obstetricians and Gynecologists (ACOG) first recommended restricted use of episiotomy in 2006 and reinforced this in 2018 [20]. Since 1996, the World Health Organization (WHO) has recommended that episiotomies be performed for fewer than 15% of births [8, 18]. National clinical practice guidelines and “norms” for the care of childbirth in Mexico appropriately describe in a non-prescriptive manner that episiotomies be performed selectively [22] and only in the context of instrumental delivery or a rigid perineum causing harm to the fetus [23].

The high prevalence of episiotomy in Mexico is simultaneously both a public health problem and a human rights imperative, since it is often carried out routinely and without a woman’s consent [14, 15]. This has spurred a number of initiatives in the last decade to improve the quality of obstetric care, with varying results [24]. This paper describes the perception of episiotomy as one element of a simulation-based training program for obstetric and neonatal emergencies, “PRONTO”, which has been replicated in Mexico, Guatemala, Kenya, Namibia and India [25]. The program uses simulation to train health care providers on teamwork, communication, emergency obstetric and neonatal care, and the application of evidence-based medicine to reduce harmful practices -including routine episiotomy [26]. PRONTO evaluations in Mexico demonstrate significant improvements in a number of beneficial practices, including the Active Management of the Third Stage of Labour (AMTSL) and delayed umbilical cord clamping. Importantly, there were no demonstrated reductions of unhelpful practices like the routine use of episiotomy in labour [26–28].

This paper presents a qualitative analysis of how health care providers perceive the practice of episiotomy both before and after they receive training in respectful maternity care. This research aims to understand how and why this practice persists despite shifts in knowledge and attitudes facilitated by participation in the PRONTO training program.

## Methods

### Study design

In 2014 and 2015, the obstetric and neonatal emergencies training program was introduced in the state of Campeche, Mexico, as a collaboration of the National Institute of Public Health (INSP) and the Women’s Institute of the State of Campeche. A qualitative study was carried out in the final year of the program to assess the perceptions of participants related to the expected outcomes, and to help explain the changes in practice, or the lack thereof. The participants who received training in 2015 worked in four community hospitals in Campeche, Mexico. The theory of behaviour change driving the intervention was based on the knowledge, attitudes and practices model [29] framed by the principles of community organization and community building [30]. This is a descriptive and interpretative qualitative study conducted both before and after a globally recognized training program in obstetric and neonatal emergencies.

### Participant selection

Participants were purposefully sampled with maximum variation [31]. We included general physicians, gynecologists and nurses at each hospital to ensure variety of the participants, help triangulate results, achieve saturation, and contribute to the richness of the data [32]. Nurses were included because the course focused on interprofessional communication, and nurses are central members of the obstetrical teams in these contexts. Inclusion criteria for participants included those directly involved in the delivery of care during labour and delivery, those selected to participate in the obstetric and neonatal emergencies training program, and those who voluntarily accepted to participate in the study. Interviews post-training were limited to participants who attended all of the components of the training sessions. Our intention was to interview the same participants before and after training. At follow up, however, some participants were ill, on vacation, had not participated, or only taken part in some of the training program. The sample thus consisted of a convenience sample of 32 individuals. We conducted a total 53 semi-structured one-on-one, face-to-face interviews (SSIs): 30 before the training (16 physicians and 14 nursing staff) and 23 interviews in follow-up, 2 of whom had not been interviewed prior to the training program (13 with physicians and 10 with nursing staff. Everyone available who met the inclusion criteria voluntarily accepted to participate in the study.

### Data collection

Two interview guides (pre and post intervention) (see Table 1), followed the main topics of the training program: perception of key evidence-based practices, perceptions of the training, utility of the training, and perceived key barriers and facilitators of changing practices. Interviews were conducted between August and September 2015 before the PRONTO training program, and in November 2015, post-intervention. SSIs were carried out at the hospitals without a third-party present to help maintain confidentiality. Interviews were audio-recorded, transcribed and lasted an average of 35 min. At the end of the interviews, analytical notes were taken to identify emerging themes and verify the saturation of the topics explored.

**Table 1:** Interview guides^a^

Interviews^b^	Themes	Questions
Baseline and Follow Up	Perception of key evidence-based practices	❖ Do you know the clinical practice guidelines or standards for emergency obstetric and neonatal care? If yes, could you mention those that seem most relevant to you? ❖ What is the use of applying oxytocin after and immediately after birth? Why do you think so? ❖ What is your opinion about the usefulness of fundal pressure (kristeller, push, boost) and in what cases should it be done? Why do you think so? ❖ **What is your opinion on the usefulness of episiotomy and in what cases should it be done? Why do you think so?** ❖ From your perspective, at what point should the umbilical cord be clamped (how long should one wait to clamp it)? Why do you think so? ❖ What is your opinion about the usefulness of uterine cleansing (cavity cleaning, cavity check) and in which cases should it be done? Why do you think so?
Only Follow-up	Perception of key evidence-based practices	❖ In the period of time between the first training module and the second module, did you participate in the delivery of one or more euthiotic births? How many? ❖ In these deliveries, were you able to apply any or all of the recommendations and/or evidence-based practices from the PRONTO program training? ➢ [Allow the informant to mention any practices he remembers. In case he does not remember, ask about: oxytocin, kristeller practice, **episiotomy**, umbilical cord clamping and cavity check]
	Perceived key barriers and facilitators of changing practices	❖ What do you think were the aspects that facilitated the implementation of such recommendations or practices? ❖ What do you think were the obstacles or barriers to carrying out such a recommendation or practice? ➢ [If not mentioned, ask about the application of oxytocin, kristeller practice, **episiotomy**, umbilical cord clamping and cavity check, and ask if any of these practices were performed on any deliveries attended]
	Perceptions of the training	❖ What do you see as the areas for improvement in the PRONTO training? (explore both content and implementation) ❖ What is your opinion about the way the trainers gave the course? How could this be improved?
	Utility of the training	❖ From your perspective, what would be the most important elements of the training PRONTO brings to your practice in childbirth and obstetric emergencies?

^a^ This table presents all the topics and questions addressed in the interview guide about the following major themes: “key practices of medical interventions during childbirth” and "general perceptions and utility of the training". However, in this article only the data concerning the topic of episiotomy are presented and discussed

^b^ The baseline interview was conducted before the implementation of the training program. The follow-up interview was performed after it

### Research team and reflexivity

The interviews were conducted by a research team member (RGC), a trained anthropologist who was not involved in the training program team. The researcher supported the evidence-based episiotomy practices and had no conflict of interest in terms of financial or professional gains in relation to the training program.

### Ethical considerations

The research protocol received ethics approval from the INSP ethics committee (project number: CI − 1196 / approval number: IRB - 1693). Data was anonymized and the confidentiality of the participants was fully protected. Participants provided consent at two stages: first they provided written consent to participate in the umbrella study of the PRONTO training program through their hospitals, with a written agreement between the Campeche Women’s Institute and the Campeche Health Ministry. We then obtained verbal consent before conducting one-on-one interviews to remind participants that their responses were anonymous and they could stop participation at any time. We provided the contact information of the research team to each of the participants.

### Data analysis

Thematic analysis was carried out [33] through axial coding of the data using the principles of grounded theory [34, 35]. The interviews and field notes were first professionally transcribed verbatim in Spanish. A codebook with codes, definitions and examples was created through an iterative process, using both the interview guide deductively and inductively to allow new codes to emerge. Atlas-ti© software [36] was used to organize the data. Once all the material was coded, the classified interviews were reread to identify any new sub-themes and findings. Three researchers (RCC, PTP, MOV) independently coded and interpreted the data. Through interpretive triangulation, the team found consensus on major themes. Any discordance was resolved through discussion and a second review of the transcripts. The findings of this article reached theoretical saturation [32]. Major themes are presented with quotes to illustrate richness and nuance of the results.

**Fig. 1:**
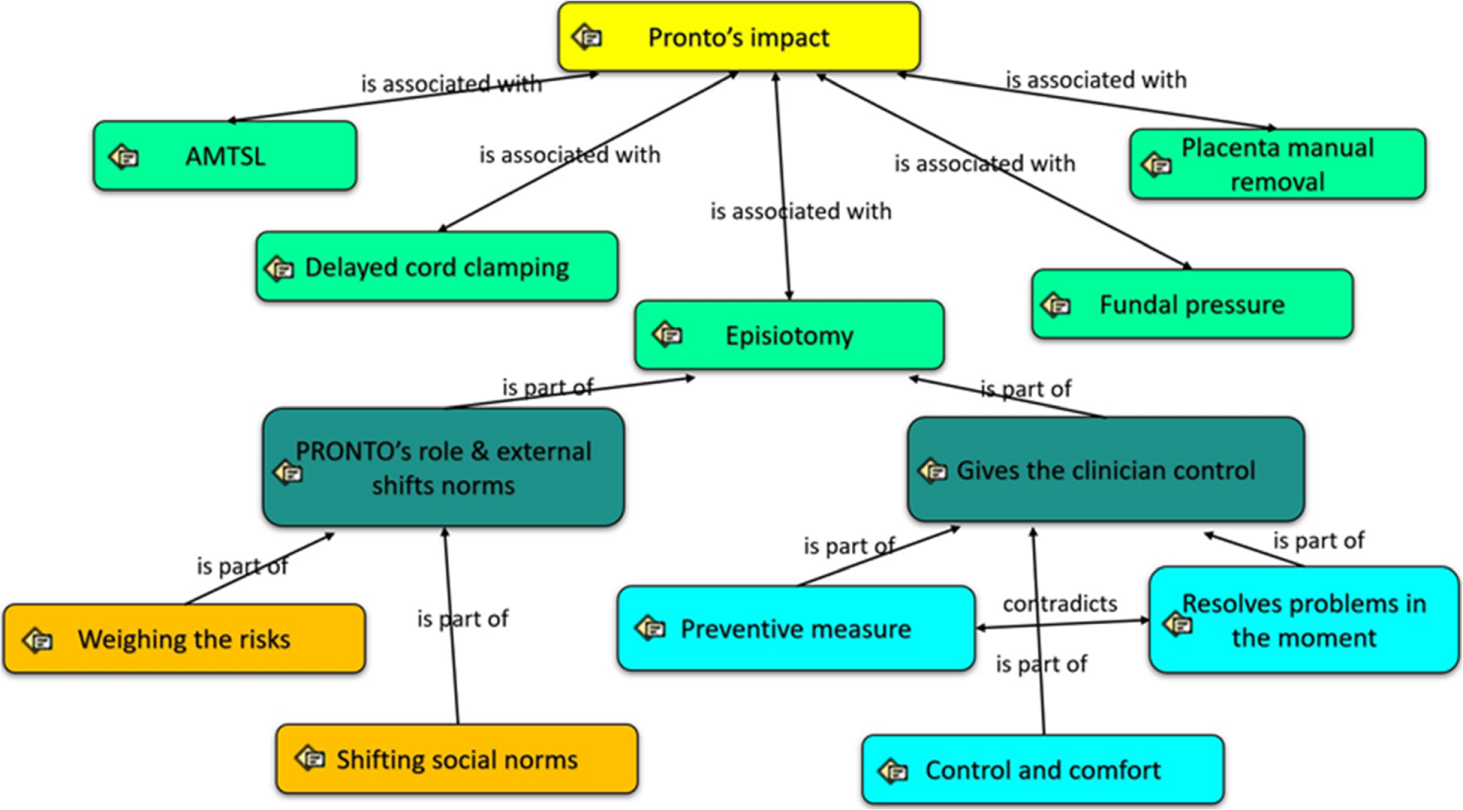
Code tree regarding to the major theme “key practices of medical interventions during childbirth”, specifying the codes analyzed from the Episiotomy topic

A total of 45 axial codes emerged under the following categories: hospital resources, work equipment, key practices of medical interventions during childbirth (episiotomy, fundal pressure, delayed cord clamping, AMTSL, and manual removal of the placenta), human rights and health and perceptions of the training program. We limited the analysis here to data pertaining to episiotomy (see Fig. 1).

## Results

A total of 32 informants participated, two thirds (66%) of whom were interviewed both before and after the intervention. The median age of the participants was 38 years old, and about half (56%) were female. The sample was comprised of 47% nursing staff, 34% generalist physicians and 19% gynecologists. Their median time working in the hospital was 8 years (see Table 2).

**Table 2:** Characteristics of the health care providers interviewed, Campeche, Mexico, 2015 (*N* = 32)

		Community Hospitals (CH)				Total
		CH1: "C"	CH2: "H"	CH3: "M"	CH4: "X"	
Average age		46	36	35	40	39
Sex	Male	4	3	4	3	14
	Female	4	4	3	7	18
Profile	Nurse	3	3	3	7	16
	Physician	2	4	1	3	10
	Gynecologists	3	0	3	0	6
Years average working at	Nurse	22	14.5	5	9.2	12.61
	Physician	16	6.25	3	5.7	7.73
	Gynecologists	11.5	–	1.3	–	6.41
Participation	Baseline	8	7	7	8	30
	Follow up	8	5	5	5	23
Interview identification codes^a^		C01, C02, C03, C04, C05, C06, C07, C08	H01, H02, H03, H04, H05, H06, H07	M01, M02, M03, M04, M05, M06, M07	X01, X02, X03, X04, X05, X06, X07, X08, X09, X10	

^a^ The Interview identification code was created with the first letter of the hospital research ID and a consecutive number. The labels that appear in the testimonies quotations were built by placing a “B” (baseline) or “F” (follow-up) followed by the interview identification code

The findings were analyzed as a single body of data, given that no substantial differences were identified between informants of different professions or hospitals. Similarly, some themes were consistent before and after the training program and thus transcripts were analyzed as a whole; where practices and perceptions had shifted or findings different post intervention, we note these findings specifically. Findings fell broadly into the following major themes surrounding different conceptions of episiotomy: the first is the overarching theme of control. Episiotomy provides the clinician a sense of control on a number of levels: as a preventive measure for women perceived as having risk factors or genetic predisposition for complications; as an effective emergency procedure that resolves problems in the moment; and for comfort with episiotomy repair. The second major theme is shifting norms and perceptions both within and outside of the training program. The obstetric and neonatal emergencies training program alongside shifts in social norms appeared to result in growing recognition that episiotomy as a practice that carries risks, despite the fact that this hasn’t translated into more restrictive use of the procedure.

### Clinician preferences versus evidence-based practices: episiotomy gives the clinician control

Overall, episiotomy was viewed positively as a tool to help prevent adverse outcomes for women categorized for genetic or medical reasons as ‘high risk’ for complications. The procedure was described as valuable for being able to prevent tears or fetal distress in a way that could be anticipated, and this was especially true for primiparous women. This framing of episiotomy as ‘prevention’ contradicted its medical value as an emergency procedure to respond to complications of labour including fetal distress - often even by the same informant. Clinicians also preferred suturing straight line episiotomy repairs. For physicians, the overall perceived risk of doing nothing outweighed the risk of the procedure.

#### Episiotomy as a preventive measure

In electing to perform an episiotomy, a number of informants both at baseline and follow up emphasized the importance of predisposing risk factors that anticipate the need for episiotomy, such as the size, weight, age and parity of the woman, as well as the weight and size of the baby:

*“The woman’s height is also a variable, usually in primiparous women who are tall as the pelvis is more suited to labor, whereas the short or skinny ones do mean more work.” General Physician, informant, B, M06*

A large or ‘macrosomic baby’ necessitated routine episiotomy as described by some informants, alongside the perception that natural tearing resulted in worse outcomes.

*‘Now we must also put ourselves from the other point of view, if the baby is large, an episiotomy has fewer complications than a third- or fourth-degree vaginal tear.” Gynecologist informant, F, M01*

“[We perform an episiotomy] *sometimes when [babies] come out macrosomic. There are times when there is no time to send [the woman] to another unit and we have to assist with the delivery anyway.” Nurse informant, F, X01*

A less common but similar theme emerged in reference to the genetic characteristics of the population predicting the need for episiotomy – described as an expected medical intervention based on ‘risk factors’.

*“[Episiotomy] is necessary much of the time. Mainly in this region of southeast [Mexico], children have super large heads, that is the reality, they are wide, they are babies with very wide shoulders, and if you do not do an episiotomy, the head comes out, but when you take out the shoulder you will tear everything.” Gynecologist informant, B, M07*

In the same way, episiotomy helped address concerns about both a woman’s risk factors and lack of prenatal care by offering a sense of control to the medical staff:

*“Those [circumstances] in which it would probably be carried out are the primips… for example, if you have not seen the patient before, she might arrive with a macrosomic baby” ... General Physician informant, B, X08*

*“There are patients who did not get any antenatal care…. Then we must face whatever comes. In this case [Episiotomy] is good because it gives us the weapons to face the things we do not expect [in order to prevent unknown problems], but it is definitely a matter of being very aware. Every woman is different.” Gynecologist informant, F, M02*

Parity was cited separately as an important factor in the decision to perform an episiotomy, but a number of contradictions emerged. Informants widely recognized that episiotomy should not be a routine practice, but the same informants state that primigravid women should systematically receive episiotomies because of the lack of elasticity of the vaginal tissue:

*“Episiotomy should not be a routine maneuver; I have had several patients in which I have not used it and I have not had any tears. Who are we going to use it on? [...] For example, in the primiparous women, whose tissues are very resistant, and there is a very high possibility of labor injuries, but there are large, multiparous women who have already had other children and in the moment I see very ‘soft’ tissue, so I consider that nothing more than guiding the head... then, specifically, I do not consider that episiotomy should be routine.” General Physician informant, B, C05*

One informant during a pre-intervention interview justified episiotomy when primiparous women “did not cooperate” with the health care providers:

*“Episiotomy is usually done to patients who are primips, who do not know or do not cooperate, that sometimes the baby is large, that is when they perform an episiotomy.” Nurse informant, B, X03*

A few informants specifically identified that there are primiparous women who do not need episiotomy, and multiparous women that do require it:

*“I have had primiparous patients who don’t need an episiotomy ... there is a small laceration, a small tear, but not something so big ... And there are patients who are multiparous and for whatever reason, we need to do an episiotomy.” General Physician informant, B, H06*

In both the pre-intervention and post-intervention interviews, informants valued these factors that help them anticipate and justify the decision to perform an episiotomy.

Episiotomy was also described as an intervention that prevents complications during childbirth. These testimonies were observed in both the baseline and follow-up interviews. We found a persistent perception that episiotomy prevents fetal distress by accelerating delivery, preventing the woman from having to push too much during the delivery and preventing the occurrence of major tears.

“[Episiotomy] *is performed when the woman, in this case, has a narrow pelvis and requires a cut for that baby to be born, as well as to avoid a tear, because then they do not stop bleeding, bleeding, bleeding...” Nurse informant, B, M04*

*“Yes I have heard cases where even if the baby can [be delivered on his own], the episiotomy is performed so as not to wait any longer.” Nurse informant, F, X09*

“[…] *You injure the pelvic floor when you do not do the episiotomy, the patient has to make much more effort and there are times when, even if you do not do the episiotomy, the baby will pass and tear everything...” Gynecologist informant, B, M07*

Two important ideas underlie these statements: the first refers to the belief that the delivery must be resolved quickly, and the second to the concept that the episiotomy accelerates and facilitates labor, preventing problems. Some informants also believed that episiotomy prevents pelvic floor disfunction and prevents significant bleeding caused by tearing:

*“[Episiotomy is] very important, always necessary to prevent pelvic floor problems, and [should be done] routinely.” Gynecologist informant, B, C06*

*“They mentioned in the course [*obstetric and neonatal emergencies training program*] that only fetal suffering is an indication for episiotomy, yes, but if you have a lady who is fully [dilated] and the baby does not come out, the tear will be worse and it will be more difficult to repair something like that ... than to repair a straight cut.” Gynecologist informant, F, M07*

Finally, another justification for performing routine episiotomy was confusion about the role of “vaginal elasticity” and confusing this with a narrow pelvic outlet. This confusion seems to have consolidated in everyday discourse and practice, although they are anatomically unrelated:

“*Sometimes, when the baby is too big, (...) even if the woman is already fully dilated, it does not come out at all, sometimes her pelvis is also very narrow and the episiotomy helps her open and there is no further complication.” Nurse informant, B, H01*

#### Episiotomy as an emergency procedure that resolves problems in the moment

In contrast to the concepts of episiotomy as either a preventative or prophylactic procedure, other informants described episiotomy as a procedure that helps resolves problems in the moment - that is, the decision about when to perform the procedure presents itself only during expulsion:

*“I believe that it is a practice that should be used, but it has its moment, when the head is crowning - there is the moment, when you have to do it.” Gynecologist informant, B, C01*

Complications during labour are taken into account in the decision to perform an episiotomy; for example, it was described as an appropriate support maneuver to relieve pressure on the cord, in the case of fetal distress or shoulder dystocia:

*“Another [circumstance] in which I could say that we could consider doing [an episiotomy] is in the looped cord, because dryness makes extraction difficult, so we need to insert the fingers (…) [The episiotomy is also indicated] in a shoulder dystocia that doesn't’ resolve despite the maneuvers ...”. Gynecologist informant, F, M02*

*“The episiotomy is only indicated in a fetal distress, if it is during expulsion.” Nurse informant, F, M03*

In the post-intervention interviews, we observed greater consistency in how informants described the circumstances by which episiotomy was appropriately used to respond to fetal distress and facilitate instrumental delivery.

#### Comfort with the procedure and perineal repair

Both in the pre-intervention and in the post-intervention interviews reflected the position that from the perspective of the clinician it is easier to repair a straight incision from an episiotomy than an irregular one caused by a natural tear, something that seems to motivate the routine use of episiotomy. This at times favored the ease of execution of the procedure, where the woman’s well-being and risks of a surgical incision were forgotten:

*“[Episiotomy] is used because sometimes when the baby's head comes out it tears the woman's perineum; it is easier to suture a cut with a scissors or something sharp, than when it is torn, because when it is torn it is already irregular ... with the scissors the cut is clean, you can suture better than when the muscle and mucosa are torn, where sometimes it reaches the rectum.” General Physician informant, B, M06*

### The role of the training in respectful maternity care and shifting norms

While this study was not an evaluation of the program per-se, interviews before and after the training and over several months allowed for some insight into the influence of the training program in shifting language, perceptions and norms. This emerged as a prominent overarching theme.

#### Weighing the risks of episiotomy

In both the pre and post-intervention interviews, a few participants were against the routine use of episiotomy, even describing it as violation of women’s bodily autonomy and a practice with limited benefits:

*“When they train us as doctors, they routinely explain to us that generally for the first birth [episiotomy] has to be performed in all women. However, I consider that this is not true, because the episiotomy, after all, is an assault (...), is a wound that is being imparted on the patient and like any other wound (...) has risks of becoming infected, and risks of opening.” Gynecologist informant, B, M01*

After participating in the training program, informants communicated a clearer perception of the risks involved in performing episiotomy, including bleeding, risk of infection and of the incision extending through the perineum:

*“If the head is crowning and we don't think there is enough room to deliver, then cut, make the incision ... but if not, it is not necessary for me to have an episiotomy, because sometimes there are doctors who go where they shouldn’t go. I see those women as very traumatized.” Nurse informant, B, C08*

*“[With episiotomy] there is a higher risk of postpartum bleeding and a higher risk of becoming infected a vaginal delivery without an episiotomy (...) If there is a tear, just repair it.” Nurse informant, F, X10*

A few informants underestimated the risk of complications, however. One informant blamed woman’s ‘lack of hygiene’ for the risk of infection with an episiotomy wound:

*“Sometimes [episiotomy] can generate a risk of infection (...) if the mother does not have the forms of hygiene and as generally, they are from here, because they do not have hygienic practices, perhaps they should wash themselves better, dry themselves well…”. Nurse informant, B, H01*

#### Shifting social norms

Social pressure emerged as one of several factors that contribute to performing episiotomy outside of clinical criteria. If there are complications such as a third degree or fourth degree tear where the episiotomy is not performed, there are social repercussions, motivating its routine use:

*“If you do not perform an episiotomy, [the vaginal canal] can be torn on several sides, and the most dangerous is when it reaches the anal sphincter; now the complication comes, which is a fistula, no? And [colleagues] are going to say ‘why didn't you do the episiotomy?’ So, the idea is that sometimes you even make a small [routine] cut and that's it.” General Physician informant, B, X08*

These statements contrast with other statements that episiotomy be avoided as a method of imparting obstetric violence and in the context of current discourse on humanized childbirth.

We also found examples where there was social pressure to *avoid* episiotomy alongside a perceived shift in norms:

*“There was a time when episiotomies were being avoided a lot ... for the same reason, because of the environment of obstetric violence, but ... well, I watched four cases of [natural] tearing ...” General Physician informant, B, H07*

*“[Episiotomy] has been avoided for humanized birth ... [delivery] has to progress… but now we say don’t push it, there is no "Be quick and done!" but rather naturally progress thus avoid episiotomy.” Nurse informant, B, H01*

Academic training and habits developed over years of clinical care were other important factors determining the practice of episiotomy. Informants shared stories of routine episiotomy being introduced during the internship period and further solidifying through clinical practice as a routine intervention:

*“Before it was everyone, even if they had a very elastic vagina ... I think that is more because we learn it this way by ...as a routine practice... when we go through internship ... but now on reflection, no it should not be performed in all cases, only in specific cases.” General Physician informant, B, H07*

Perceptions also shifted after the obstetric and neonatal emergencies training to be generally more supportive of selective episiotomy, voicing more personal conviction in implementing the recommendations of the training program.

*“On two occasions I did not do the episiotomy and the tears were minimal. Everything was fine, we weren't used to not doing episiotomy [before the training], but I already put it into practice.” General Physician informant, F, M06*

One positive outcome of the program was a shift in post intervention informants describing the procedure as necessary, but only in some cases:

*“ I liked the section on episiotomy in the course , because it reiterated that that sometimes it can be omitted (...) in particular cases. There are some cases in which it is very well indicated and necessary.” Gynecologist informant, F, C06*

Several informants in post-intervention reflected shifting perceptions about the delivery care, which was more focused on the well-being of women and supporting her in labor:

*“I think that the important thing here is to assess each patient because you also have to have a connection with the patient and you have to know how to support her well. A well-monitored, well supported labor will support a naturally progressing labour where you see that everything is going well, and can avoid an episiotomy.” Gynecologist informant, F, M01*

## Discussion

This study provides unique perspective into health care provider perceptions surrounding episiotomy in a high prevalence setting. For the first time in Mexico, qualitative methods reveal the context for the research-practice gap, and why this routine medical practice persists despite well described harms to the health of women. This study highlights why routine episiotomy remains a stubborn medical practice to change [21]. Attitudes and beliefs regarding episiotomy are based on subjective criteria of clinicians that, far from corresponding to scientific evidence, prioritize the preferences of health care providers over the well-being of the woman. This practical insight needs to translate into effective interventions to close the gap between evidence and practice [37] for this “Too Much Too Soon” intervention [7]. Joseph B. DeLee’s outdated recommendations still persist in practice [38].

We observed that some clinicians have a clearer conception of the risks of episiotomy after training in respectful maternity care, despite lack of consensus on when it should be performed. A few informants even described social pressure to *avoid* episiotomy in the context of the global movement towards respectful maternity care. These attitudes have yet to translate to more selective use of episiotomy which remains enshrined with positive attributes for its power to prevent anticipated complications of childbirth.

Routine episiotomy is a global problem, and similar rationale for routine episiotomy have been described in other studies. Both qualitative and quantitative studies worldwide identified that parity and the anatomical characteristics of the woman and fetus influenced how clinicians perceived the need for episiotomy, which is directly linked to fear of tearing [39–41]. Perhaps unique to our study is the finding that physicians conflate pelvic size and perineal elasticity. Overall we observed persistent, positive view of episiotomy as a practice that reassures providers, and resolves anticipated complications based on the routine nature of the practice [37]. Post-training, informants still viewed episiotomy positively to help accelerate birth, reduce maternal effort, and prevent complications, congruent with a 20 year old study by Klein et al. [42].

Attitudes and beliefs surrounding episiotomy appear particularly subjective, and clinician preferences remained misaligned with evidence-based practice. Seijmonsbergen-Schermers and colleagues drew very similar conclusions in their recent qualitative study [43]. Perhaps the most striking example of this subjectivity is a study which randomized patients to receive restricted versus routine episiotomy: their clinical opinion remained the most important factor in deciding to perform episiotomy [44, 45]. One third of physicians didn’t change their practice, and refused to open the study envelope, using episiotomy 90% of the time in both trial arms. Physicians who viewed episiotomy more positively were more likely to view normal labour as abnormal – overestimating fetal distress or underestimating the ability of the perineum to stretch.

Another study found that fewer than one-third of obstetricians surveyed relied on evidence-based literature in their decision to perform episiotomy [46]. One striking example of this from ours and several other studies is that clinicians stated preference to suture a straight cut over spontaneous perineal tears, despite the literature that suggests that an episiotomy requires equal or more time to repair [47–49]. Moreover, it does not prevent tears in normal deliveries [50]. In this sense, routine episiotomy remained normalized and grounded in early training, where their supervisors and mentors performed routine episiotomies. Teachers may not have learned how to repair three-dimensional natural tears or feel comfortable doing so [42, 51, 52]. This has significant implications for training and behavior change; early and continuous medical education training should include three-dimensional suturing models [20, 53], and identify a local leader who can mentor other physicians [25, 26].

This study also highlights the unique challenges for changing a routine practice that has potential benefits, as compared to a routine test or procedure that is harmful or has no benefits. This nuance is reflected in a recent cohort study which confirmed that episiotomy is protective against anal sphincter injury in operative (vacuum or forceps) vaginal delivery [54]. The potential for benefit makes episiotomy particularly difficult practice to change, regulate, and ‘control’.

National guidelines in both middle- and high-income countries endorse the restricted, or selective, use of episiotomy, but leave room for the practitioner to use their discretion. Clinical guidelines from the American College of Obstetricians and Gynecologists (ACOG) in the USA and the National Institute of Clinical Evidence (NICE) in the UK both recommend against the routine use of episiotomy, but leave ample room for clinician discretion stating that physicians should use their clinical judgment to decide when the procedure is necessary [55, 56]. It remains unclear how much of the decline in episiotomy in the US can be attributed to the publication of the ACOG guidelines: at that time, one fourth of all vaginal deliveries resulted in episiotomy, but it’s use was already in decline, down from 34% [19].

Urban nonteaching hospitals having the highest rates of episiotomy, a finding consistent with several studies showing significant differences depending on the teaching status of the hospital [57–59]. The publication of the ACOG clinical guidelines precipitated a narrowing of this discrepancy, and thus may have helped reduce disparities between institutions [60]. Another factor is how much confidence and trust is placed in governing bodies and national guidelines [46]. Dramatic decreases in the episiotomy rate in France were attributed to the College of Obstetricians and Gynecologists (CNGOF) because its clinical practice guidelines clearly advocated for a policy of restrictive episiotomy [61, 62]. In contrast, the wording of the 2016 ‘Official Norm’ for Mexico remains purposefully vague and supports the provider to use their clinical judgement [22]. The ‘clinical guideline for the care of women in labour’ is also thought to exert little influence in Mexico because it is not enforced either positively or punitively [23]. Globally, high quality guidelines are necessary, but not sufficient to close the “know-do gap” [7, 63].

Considering this, and the particularly complex, ‘stubborn’ and ritualistic nature of routine episiotomy, what has worked elsewhere at a facility, programmatic, and population-level? At the facility level, restrictive episiotomy hospital policies were successful in dramatically decreasing the episiotomy rate in both Nepal [64] and Hong Kong [65]. Zhang-Rutledge and colleagues [66] took a quality improvement approach with success: they used education, performance feedback, and the Hawthorne effect with a significant reduction in the episiotomy rate in a large academic institution. At a programmatic and population level, there is less data to support episiotomy practices specifically. We do know generally that guidelines are more likely to be implemented with a mix of audit and feedback, financial incentives, simulations and drills, and continuing professional development [67–70]. Programs and interventions that only target providers are likely to have limited effect [71].

A systematic approach to preventing harmful obstetrical practices would directly engage women and communities. One indicator of respectful maternity care, for example, is offering birth companions to women in labour, which is strongly encouraged by the WHO [72]. This unique combination of support and advocacy during labour might may indirectly impact harmful and abusive practices during childbirth [73]. Particularly important is the approach of these respectful practices in obstetric care in universities, in order to base teaching on scientific evidence. The role of medical schools in teaching appropriate, restricted use of episiotomy and other respectful practices during childbirth is a major topic for future research.

Episiotomy was not the only focus on the obstetric and neonatal emergencies training program, whose main objective was to provide training in emergency obstetric care. However, we suspect that given shifts in knowledge and attitudes post-training that a more comprehensive approach -including facility-level guideline development, local leadership, supervision, and support- may have effective to move practice.

Our study has some limitations. Initially, not all subjects who participated in the baseline study were able to participate post-training. However, the objective of this qualitative study was not to complete a formal course evaluation, but rather understand the perceptions behind the practices of the medical and nursing staff. Indeed, one of the strengths of the study is having interviewed almost all of the subjects trained in the program before and after receiving the training, allowing for some inference about the early impact of the training itself.

## Conclusions

Routine episiotomy remains a particularly stubborn practice to change, positively perceived as effective prophylaxis for the complications of childbirth while maintaining control in the hands of physicians.

Physicians maintain a positive perception of episiotomy – where intervention is valued over patience and they fear repercussions of not intervening. They are mentored and trained to perform routine episiotomies and feel more comfortable with their repair. Social pressure to perform episiotomy continues to be the mechanism by which this practice is perpetuated in schools and hospitals, despite current scientific evidence. This vision of care is what influences practice. Post-training, informants still viewed episiotomy positively to help accelerate birth, reduce maternal effort, and prevent complications, but had a more awareness of the potential harms to women.

When we look at the global movement in respectful maternity care, single trainings are not enough to change “Too Much Too Soon” obstetric practices. Facility-level quality improvement programs and national guidelines should be met with bottom-up approaches to supporting respectful maternity care with birth companionship. The national practice guidelines in Mexico may be too ambiguous to help move this agenda forward. To close the research-policy-practice gap, we urgently need multifaceted approaches which combine clear, high quality guidelines with quality improvement approaches to supervision and support, audit and feedback, simulations and drills, and continuing professional development.

## Data Availability

The dataset generated (interviews’ transcripts) and analyzed during the current study is not publicly available due to the fact that the data would be sufficient to identify individual informants and, therefore, violate their confidentiality. However, it can be requested from the corresponding author who can provide anonymized data on reasonable request. It is only available in Spanish.
